# Whole-Genome sequencing and genetic variant analysis of a quarter Horse mare

**DOI:** 10.1186/1471-2164-13-78

**Published:** 2012-02-17

**Authors:** Ryan Doan, Noah D Cohen, Jason Sawyer, Noushin Ghaffari, Charlie D Johnson, Scott V Dindot

**Affiliations:** 1 Department of Veterinary Pathobiology, Texas A&M University College of Veterinary Medicine and Biomedical Sciences, College Station, TX, USA; 2 Department of Large Animal Clinical Sciences, Texas A&M University College of Veterinary Medicine, College Station, TX, USA; 3 Texas AgriLife Research Center, McGregor, TX, USA; 4 Department of Animal Science, Texas A&M University, College Station, TX, USA; 5 AgriLife Genomics and Bioinformatics Center, College Station, TX, USA; 6 Department of Molecular and Cellular Medicine, Texas A&M Health Science Center College of Medicine, College Station, TX, USA

## Abstract

**Background:**

The catalog of genetic variants in the horse genome originates from a few select animals, the majority originating from the Thoroughbred mare used for the equine genome sequencing project. The purpose of this study was to identify genetic variants, including single nucleotide polymorphisms (SNPs), insertion/deletion polymorphisms (INDELs), and copy number variants (CNVs) in the genome of an individual Quarter Horse mare sequenced by next-generation sequencing.

**Results:**

Using massively parallel paired-end sequencing, we generated 59.6 Gb of DNA sequence from a Quarter Horse mare resulting in an average of 24.7X sequence coverage. Reads were mapped to approximately 97% of the reference Thoroughbred genome. Unmapped reads were *de novo *assembled resulting in 19.1 Mb of new genomic sequence in the horse. Using a stringent filtering method, we identified 3.1 million SNPs, 193 thousand INDELs, and 282 CNVs. Genetic variants were annotated to determine their impact on gene structure and function. Additionally, we genotyped this Quarter Horse for mutations of known diseases and for variants associated with particular traits. Functional clustering analysis of genetic variants revealed that most of the genetic variation in the horse's genome was enriched in sensory perception, signal transduction, and immunity and defense pathways.

**Conclusions:**

This is the first sequencing of a horse genome by next-generation sequencing and the first genomic sequence of an individual Quarter Horse mare. We have increased the catalog of genetic variants for use in equine genomics by the addition of novel SNPs, INDELs, and CNVs. The genetic variants described here will be a useful resource for future studies of genetic variation regulating performance traits and diseases in equids.

## Background

The sequencing and assembly of a horse genome was a great achievement in equine genomics and veterinary medicine because of the broad range of potential applications of this information for improving health and performance and for understanding differences among species [1]. To date, however, only a single genome of a Thoroughbred mare has been sequenced and made publicly available [1]. The current catalog of genetic variants in the equine genome consists of 1,163,580 single nucleotide polymorphisms (SNPs; dbSNP: http://www.ncbi.nlm.nih.gov/projects/SNP/ [build 135]), with no insertion/deletion polymorphisms (INDELs) or copy number variants (CNVs) having been deposited into a publicly available database (dbSNP or dbVar, http://www.ncbi.nlm.nih.gov/dbvar). Of the known SNPs in horses, most (~64%) originate from the Thoroughbred mare used for the genome assembly [1].

Sequencing the genome of a Quarter Horse was considered important for several reasons. Almost 3 million Quarter Horses were registered in the United States in 2010 according to the American Quarter Horse Association, making it the single largest breed registry in the country [2,3]. When one includes non-registered Quarter Horses and other Quarter Horse-influenced breed registries (e.g., American Paint Horse Association), the Quarter Horse is by far the largest contributor to the population of horses in the United States. Although Quarter Horse breeding was, and continues to be, strongly influenced by Thoroughbred bloodlines, the 2 breeds were selectively bred to enhance different traits. Thoroughbred breeding has selected for speed over distances of 3/4 to 2 miles, whereas selection in the Quarter Horse has emphasized speed over shorter distances and compliant disposition suited to working cattle and other ranch-related duties [4]. Thus, there are important phenotypic differences between the breeds that have been achieved by selective breeding with a clear underlying genetic component [5,6]. Moreover, there are single-gene disorders in Quarter Horses, such as polysaccharide storage myopathy (PSSM) [7,8], hyperkalemic periodic paralysis (HYPP) [9], glycogen branching enzyme deficiency (GBED) [10], and hereditary equine regional dermal asthenia (HERDA) [11,12]. Most diseases and traits (such as predisposition to osteoarthritis or body-type) are complex, involving multiple genes, which may be modulated by environmental factors [5,13,14]. Thus, identifying genetic variants in the genome of a single Quarter Horse would provide a wealth of information for future genomic studies in equids.

Here we describe the whole-genome sequencing of an individual Quarter Horse mare using massively parallel paired-end sequencing. Sequence reads were mapped to the reference Thoroughbred nuclear and mitochondrial genomes. We developed a comprehensive list of genetic variants, including SNPs, INDELs, and CNVs. We annotated genetic variants and examined their impact on gene structure and function. The genomic sequence was also examined for mutations and polymorphisms associated with diseases and traits in horses. Furthermore, we examined biological processes enriched for genetic variants and compared these biological processes between this Quarter Horse mare and the reference Thoroughbred mare.

## Results

### Whole-genome sequencing, alignment, and identification of new genomic sequence

**Figure 1:**
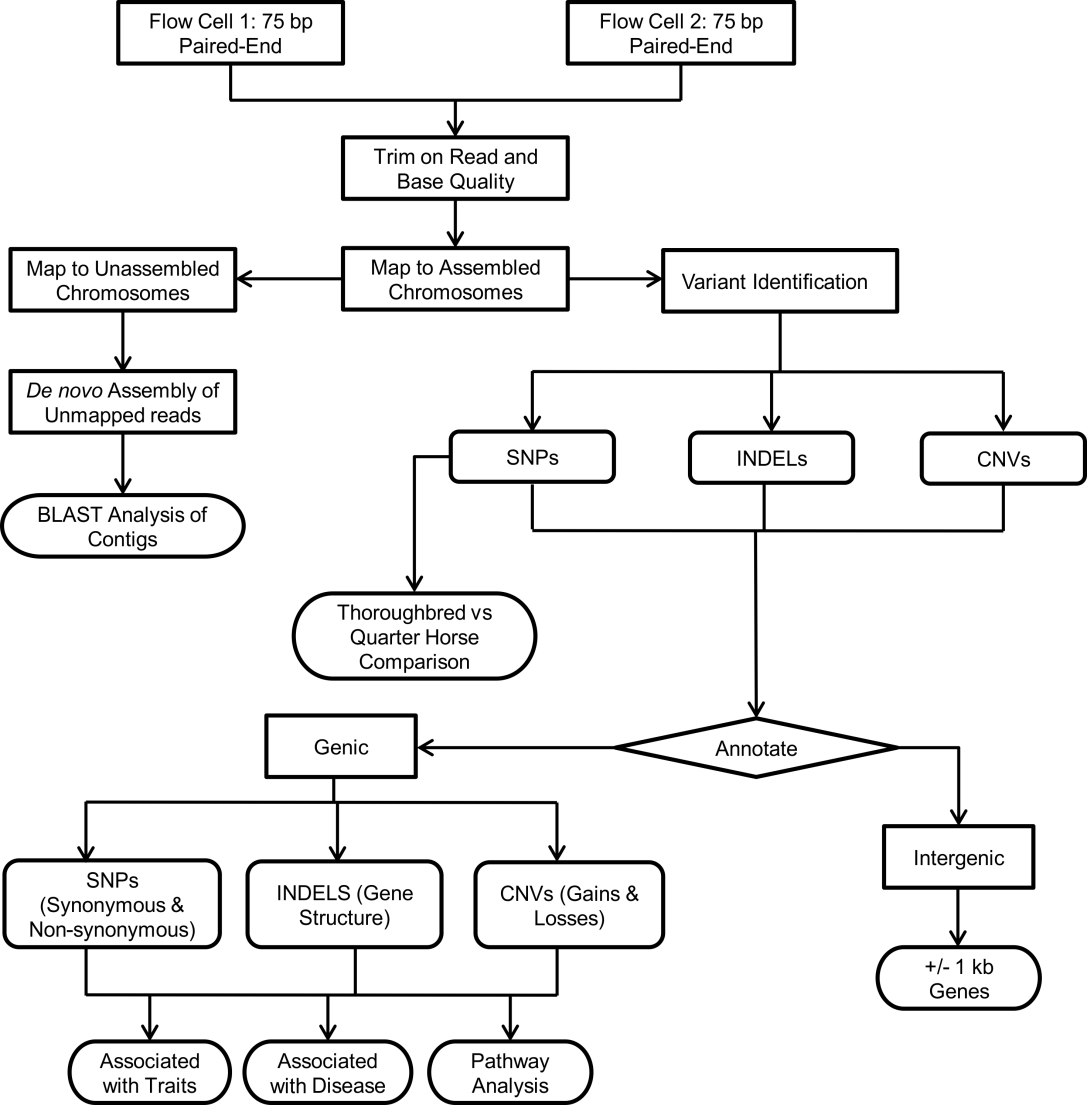
**Overview of genome-wide variation analysis pipeline by next generation sequencing**.

Genomic DNA was obtained from a single blood sample of a Quarter Horse mare owned by Texas A&M University. The mare's pedigree was heavily influenced by stock and racing Quarter Horses, with no introgression of Thoroughbred lines during the preceding 4 generations. A small insert library with an average size of approximately 270 base-pair (bp) was generated (Additional file 1, **Figure S1**), and 14 lanes of 75-bp paired-end sequencing were performed using the Illumina Genome Analyzer II (Additional file 2, **Table S1**) (Illumina, Inc., San Diego, CA). A schematic of the sequence mapping and variant identification, annotation, and analysis is presented in Figure 1. The sequencing reactions yielded 849,600,742 reads totaling 61,310,282,925 bases of DNA (Additional file 3, **Table S2)**. Sequence reads were trimmed and mapped to the assembled autosomes, × chromosome, and mitochondrial genome of the reference Thoroughbred horse genome (EquCab2.0). A total of 817,470,439 reads (59,643,456,282 bases) were aligned to approximately 97% of the reference genome (53,783,377,648 bases were uniquely mapped), resulting in an average of 24.7X sequence coverage of the horse's genome (Additional file 3, **Table S2)**. Sequence coverage of assembled chromosomes ranged from 13X to 55X (Additional file 4, **Figure S2**). Additionally, 81,993 reads mapped to 100% of the reference mitochondrial genome, resulting in an average of 355.6X sequence coverage. Reads not mapping to assembled chromosomes were subsequently mapped to the unassembled chromosomes (ChrUn), where 12,865,623 reads (935,090,584 bases) were mapped. Next, we *de novo *assembled 12,657,236 reads (707,253,380 bases) not mapping to the assembled or unassembled chromosomes. The *de novo *assembly yielded 35,540 contigs totaling 19.1 million bases (Mb) of sequence (Table 1). BLAT and BLAST analysis of the contigs to other genomes did not result in any significant alignment.

**Table 1:** Sequence generation and mapping to equCab2 reference genome

Method	Lanes	Reads	Bases Mapped (Gb)	Average Depth of Coverage	% of Reference Mapped	Bases Mapped to ChrUn (Mb)	*De Novo *Assembly of Unmapped (Mb)
75 PE	14	817,470,439	59.6	24.7 X	97%	935.1	19.1

### Identification of genetic variants

We analyzed sequences mapped to the assembled chromosomes for putative SNPs, INDELs, and CNVs. SNPs were required to meet several stringent criteria (see **Materials and Methods**) and were further filtered to remove those near other variants (i.e., SNPs and INDELs) or within the pseudo-autosomal region (PAR) of the × chromosome. The resulting SNPs were then filtered using different minimum read-depths (5X and 10X [Additional file 5, **Table S3 and **Additional file 6, **Table S4]**). As expected, increasing the minimum read-depth resulted in fewer SNPs being detected (1,131,519 SNPs lost from 5X to 10X coverage; Additional file 7, **Table S5 and **Additional file 8, **Figure S3A and B**). Additionally, we found that a greater proportion of the total SNPs was lost than that of the total non-synonymous SNPs, with homozygous SNPs being lost at the highest rate (Additional file 8, **Figure S3C and D**). At 10X coverage, which was the minimum read-depth used for the subsequent annotation and downstream analysis, we identified 3,157,093 SNPs. Additionally, we characterized and annotated SNPs detected at lower minimum read-depths (Additional files 9, 10, 11, 12, 13, 14, 15, 16, 17, 18, 19, 20, 21, 22, and 23, **Tables S6, S7, S8, S9, S10, S11, S12, S13, S14, S15, S16, S17, S18, S19, and S20)**. Intergenic SNPs, located within 1,000 bases (1 kb) upstream or downstream of a gene, were determined as well as genic SNPs located within introns, non-coding exons, 5' and 3' untranslated regions (UTR), intron and exon splice sites, and coding exons. Non-synonymous SNPs were further annotated as either radical or conservative (Table 2, **and **Additional files 9, 10, 11, 12, 13, 14, 15, 16, 17, 18, 19, 20, 21, 22, and 23, **Tables S6, S7, S8, S9, S10, S11, S12, S13, S14, S15, S16, S17, S18, S19, and S20**; see **Materials and Methods **for description of amino acid classification). Additionally, we identified SNPs located within the coding exons of 305 candidate imprinted genes for future studies in the horse ([15]; Additional file 24, **Table S21**). Analysis of the mitochondrial genome identified 71 SNPs, including 5 heteroplasmic SNPs (Additional file 5, **Table S3 and **Additional file 6, **Table S4**).

**Table 2:** Annotation of SNPs in the Quarter Horse genome

	Total	Homozygous	Heterozygous	Novel	Ensembl Genes
**All SNPs**	3,157,093	1,293,374	1,863,719	2,814,367	24,023
**Intergenic**	2,224,292	908,169	1,316,123	1,991,041	19,644
**Intergenic (Upstream w/in 1 kb)**	40,948	19,241	21,707	37,566	12,803
**Intergenic (Downstream w/in 1 kb)**	31,901	13,327	18,574	28,494	10,580
**Intergenic (Up/Down w/in 1 kb)**	929	427	502	866	399
**Genic**	859,023	352,210	506,813	756,400	19,414
**Intron**	811,204	332,455	478,749	712,764	14,778
**Non-Coding Exon**	5,935	2,019	3,916	5,575	2,123
**5' UTR**	1,734	1,014	720	1,630	1,049
**3' UTR**	1,821	780	1,041	1,594	1,184
**Intron Splice Site**	300	185	115	280	276
**Exon Splice Site**	657	320	337	593	595
**Coding Exon**	37,372	15,437	21,935	33,964	10,485 (305 imprinted)
**Synonymous**	19,667	7,626	12,041	17,699	7,982
**Stop Gain**	214	50	164	206	190
**Stop Loss**	8	5	3	7	8
**Non-Synonymous**	18,140	8,076	10,064	16,645	6,899

Comparison of putative SNPs in the mapped sequences to the horse SNP database (dbSNP, http://www.ncbi.nlm.nih.gov/projects/SNP/) revealed that 342,726 were known and 2,814,367 were novel, including 18,140 non-synonymous SNPs (11,434 radical and 6,706 conservative) and 2,629 complex (i.e., tri-allelic) SNPs. Comparison of the putative SNPs to those identified in the sequencing and assembly of the reference Thoroughbred genome revealed that 244,669 SNPs were overlapping between the 2 horses, including 174,682 intergenic and 69,987 genic SNPs. By removing overlapping known SNPs, we found that the Quarter Horse and Thoroughbred genomes had 2,912,424 and 522,610 unique SNPs, respectively.

Using a minimum read-depth of 10X and similar filtering methods described for the SNP analysis, we identified 193,271 INDELs (1-8 bp insertion or deletions; Table 3**and **Additional file 25, **Table S22**). Additionally, we identified and annotated INDELs using lower read-depth coverage (5X; Additional file 26, **Table S23**). Although our analysis was limited to INDELs no greater than 8 bp, the overwhelming majority were single bp gains and losses (Additional file 27, **Figure S4**). Of the INDELs identified, 2,574 caused a frameshift mutation (frameshift deletion + insertions) in a coding exon, whereas 35 did not (Table 3). In addition, 21 INDELs lead to the creation of a stop codon (stop - gain). Given the high number of INDELs affecting coding exons, particularly the large number of homozygous INDELs, we annotated them using the RefSeq annotation. The analysis revealed that 50 RefSeq genes contained INDELs, including 7 genes with loss of a stop codon and 47 with a frameshift.

**Table 3:** Annotation of INDELs in the Quarter Horse genome

INDEL Location	Total	Homozygous	Heterozygous
**INDELs (1 bp - 8 bp)**	193,271	127,173	66,098
**Intergenic**	122,571	78,538	44,033
**Intergenic (Upstream w/in 1 kb)**	5,900	5,073	827
**Intergenic (Downstream w/in 1 kb)**	2,557	1,853	704
**Intergenic (Up/Down w/in 1 kb)**	126	105	21
**Genic**	62,117	41,604	20,513

**Intron**	57,618	37,377	20,241
**Non-Coding Exon**	277	239	38
**5' UTR**	594	557	37
**3' UTR**	249	195	54
**Intron Splice Site**	749	735	14
**Exon Splice Site**	771	762	9
**Coding Exon**	1,859	1,739	120

**Frameshift Deletion**	1,594	1,533	61
**Frameshift Insertion**	980	940	40
**Non-Frameshift Deletion**	23	4	19
**Non-Frameshift Insertion**	12	5	7
**Stop - Gain**	21	19	2

Copy number variants (CNVs) were identified using a read-depth algorithm that corrects for GC bias. CNVs were then filtered to remove those within telomeric regions, as investigation of these regions is associated with high false discovery rates [16]. The filtered analysis revealed 282 CNVs, including 274 gains, 6 losses, and 2 homozygous deletions (Additional file 28, **Table S24**, Additional file 29, **Figure S5 and **Additional file 30, **Figure S6**). The sizes ranged from 3.74 kb to 4.84 Mb, with an average length of 296.1 kb. Annotation of the CNVs indicated that 192 and 90 were genic and intergenic, respectively.

### Pathway, trait, and disease analysis of identified genetic variants

**Figure 2:**
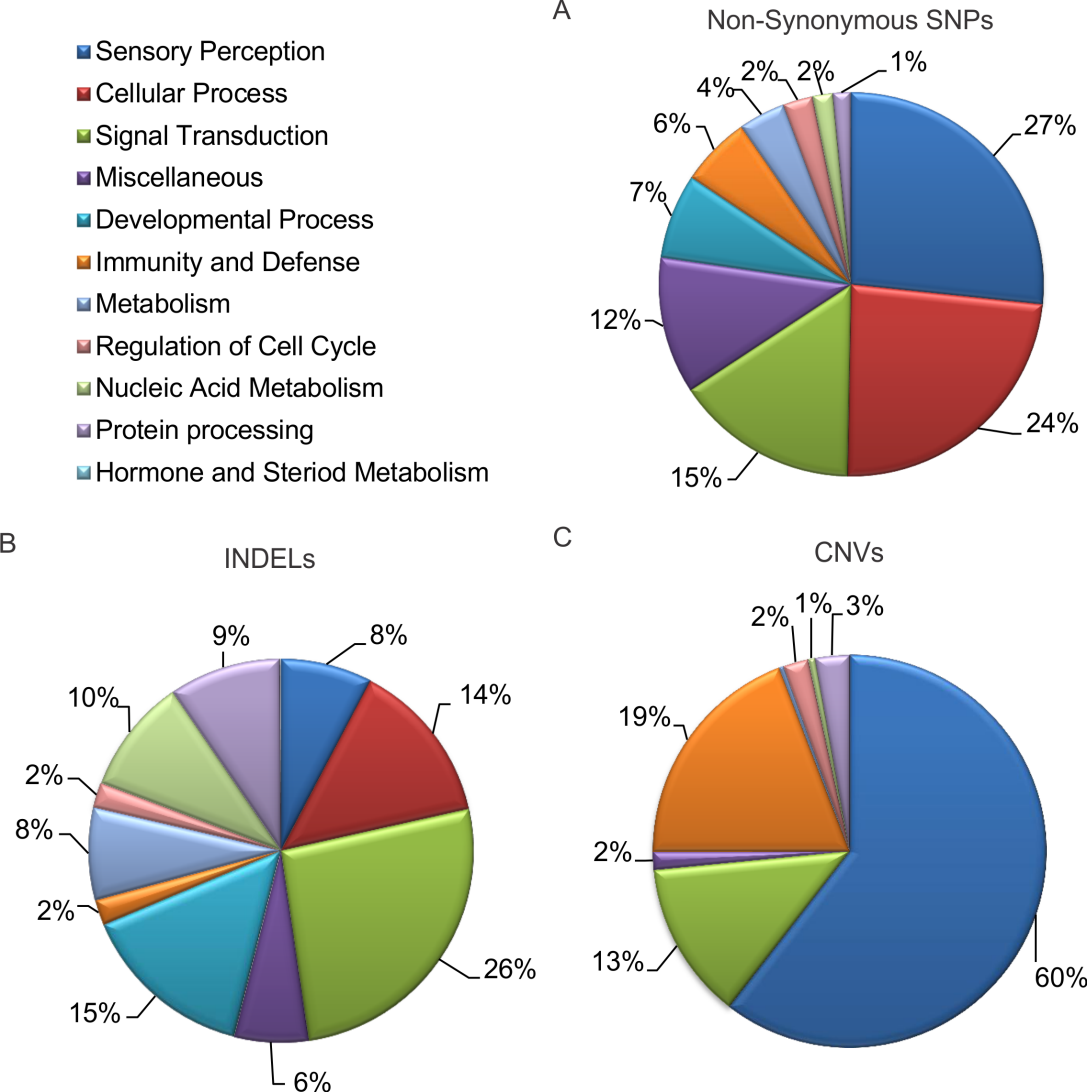
**Biological process enrichment analysis of identified genetic variants**. (A) BP enrichment analysis of non-synonymous coding SNPs, (B) INDELs, and (C) CNVs.

We performed a functional annotation clustering analysis of genes containing SNPs, INDELs, and CNVs to identify biological processes (BP) enriched for these classes of genetic variants. Using the horse Ensembl gene identification (ID) for the functional annotation clustering, we found that only a small percentage of BP terms were returned (0.6%). Therefore, we decided to perform the analysis using the human orthologs of horse genes found to possess genetic variants. Of the 6,899 genes containing non-synonymous SNPs, 5,712 had human orthologs and 3,880 returned BP terms (67.9%). Clustering analysis indicated that non-synonymous SNPs were enriched (P < 0.05) in pathways for sensory perception (27%; P = 3.9 × 10^-15^), cellular processes (24%; P = 1.7 × 10^-6^), and signal transduction (16% P = 2.7 × 10^-7^; Figure 2A and Additional file 31, **Table S25**). Separate functional enrichment analysis of genes with either radical or conservative non-synonymous SNPs revealed that the biological processes of the enriched pathways remained unchanged, although there was an increase in enrichment of conservative amino acid changes in the immunity and defense pathway (3% radical and 14% conservative; Additional file 32, **Figure S7A and B**). Clustering analysis of INDELs indicated that genes containing variants were enriched in pathways for signal transduction (26%; P = 4.2 × 10^-6^), developmental processes (15%; P = 3.5 × 10^-10^), and cellular processes (14%; P = 1.4 × 10^-5^; Figure 2B). Analysis of CNVs indicated that genes containing variants were enriched in pathways for sensory perception (60%; P = 2.1 × 10^-61^), immunity and defense (19%; P = 2.7 × 10^-2^), and signal transduction (13%; P = 1.3 × 10^-3^; Figure 2C).

Comparison of biological pathways containing heterozygous non-synonymous SNPs between the Quarter Horse and reference Thoroughbred (Additional file 33, **Table S26) **revealed that the Quarter Horse had SNPs enriched in pathways for sensory perception (36%; P = 1.3 × 10^-36^), cellular processes (18%; P = 4.8 × 10^-5^), and signal transduction (16%; P = 1.2 × 10^-3^; Additional file 34, **Figure S8A**), whereas the Thoroughbred had SNPs enriched in pathways for immunity and defense (36%; P = 5.2 × 10^-8^), sensory perception (23%; P = 6.4 × 10^-14^), and cellular processes (13%; P = 2.7 × 10^-1^; Additional file 34, **Figure S8B**). As the enrichment analysis reflected percentages of genes within pathways, and because the Quarter Horse contained nearly three times as many heterozygous SNPs as the Thoroughbred, we determined the total number of genes for each BP term that contained a non-synonymous heterozygous SNP. As expected, the Quarter Horse had more genes containing SNPs for each BP class, except for the immune and defense (P = 2.4 × 10^-27^) and nucleic acid metabolism (P = 1.1 × 10^-11^) pathways (Additional file 34, **Figure S8C**, Additional file 35, **Tables S27 and **Additional file 36, **Table S28**).

Next, we investigated the DNA sequence of the Quarter Horse mare for mutations of known diseases and for SNPs associated with various traits in horses (Table 4). We found the Quarter Horse to be heterozygous for a c.115G > A mutation in the cyclophilin B (*PPIB*) gene, which causes hereditary equine regional dermal asthenia (HERDA)[12], heterozygous for the g.901C > T SNP associated with chestnut coat color [17], and homozygous for the g.66493737C > T and g.22684390C > T SNPs associated with racing endurance in Thoroughbred horses [18,19]. No other mutations or polymorphisms underlying known diseases or traits were identified.

**Table 4:** Analysis of genetic variants for known traits and diseases

PMID	Chromosome	Coordinate	Gene	Gene Name	Phenotype	Associated Genotype	QH Genotype
20353955 [32]	1	108,249,293	*TRPM1*	transient receptor potential cation channel	Leopard complex spotting and congenital stationary night blindness	C/C C/T	T/T
17498917 [12]	1	128,056,148	*PPIB*	peptidyl-prolyl cis-trans isomerase B	Hereditary equine regional dermal asthenia	A/A	G/**A**
20419149 [33]	1	138,235,715	*MYO5A*	myosin-Va isoform 1	Lavender foal syndrome	Del 1 bp	Neg
8995760 [34]	3	36,259,552	*MC1R*	melanocyte-stimulating hormone receptor	Chestnut coat color	T/T	C/**T**
11086549 [17]	3	36,259,554	*MC1R*	melanocyte-stimulating hormone receptor	Chestnut coat color	A/A	G/G
16284805 [35]	3	77,735,520	*KIT*	mast/stem cell growth factor receptor	Sabino spotting	A/A A/T	T/T
18253033 [36]	3	77,740,163	*KIT*	mast/stem cell growth factor receptor	Tobiano spotting pattern	A/A A/G	G/G
21070277 [6]	4	38,969,307	*PDK4*	pyruvate dehydrogenase kinase isozyme 4	Racing performance	A/A A/C	C/C
21070277 [6]	4	38,973,231	*PDK4*	pyruvate dehydrogenase kinase isozyme 4	Racing performance	A/A A/G	G/G
12230513 [37]	5	20,256,789	*LAMC2*	laminin subunit gamma-2 precursor	Junctional epidermolysis bullosa	Ins C	Neg
17029645 [38]	6	73,665,304	*PMEL17*	melanocyte protein 17 precursor	Silver coat color	T/T T/C	C/C
19016681 [39]	8	45,603,643-45,610,231	*LAMA3*	laminin alpha-3	Junctional epidermolysis bullosa	Del 6,589	Neg
9103416 [40]	9	35,528,429	*DNAPK*	DNA-dependent protein kinase catalytic subunit	Severe combined immunodeficiency	Del 5 bp	Neg
15318347 [41]	10	9,554,699	*RYR1*	ryanodine receptor 1 isoform 2	Malignant hyperthermia	G/G G/C	C/C
21059062 [19]	10	15,884,567	*CKM*	creatine kinase M-type	Racing performance	A/A A/G	G/G
18358695 [7]	10	18,940,324	*GYS1*	glycogen [starch] synthase muscle	Polysaccharide storage myopathy	A/A A/G	G/G
7623088 [42]	11	15,500,439	*SCN4A*	sodium channel protein type 4 subunit alpha	Equine hyperkalemic periodic paralysis	G/G G/C	C/C
18802473 [43]	14	26,701,092	*SLC36A1*	proton-coupled amino acid transporter 1	Champagne dilution	C/C C/G	G/G
9580670 [44]	17	50,624,658	*EDNRB*	endothelin B receptor precursor	Lethal white foal syndrome	AG/AG	TC/TC
20932346 [18]	18	66,493,737	*MSTN*	growth/differentiation factor 8 precursor	Optimum racing distance	T/T	**T/T**
12605854 [45]	21	30,666,626	*SLC45A2*	membrane-associated transporter protein isoform	Cream coat color	A/A A/G	G/G
21059062 [19]	22	22,684,390	*COX4I2*	cytochrome c oxidase subunit 4 isoform 2	Racing performance	T/T T/C	**T/T**
11353392 [46]	22	25,168,567	*ASIP*	agouti-signaling protein precursor	Black and bay color	Del 11 bp	ND
18641652 [47]	25	6,574,013-6,581,600	*STX17*	syntaxin 17	Gray coat color	Dup 4,600	Neg

Abbreviations: Del, Deletion; Ins, Insertion; Neg, Negative; ND, Not Determined; Dup, Duplication

## Discussion

The current catalog of equine genetic variants is limited and primarily consists of those detected from the sequencing and assembly of a single Thoroughbred horse [1]. In the present study, we describe the whole-genome sequencing and identification of genetic variants in the genome of a Quarter Horse mare. To our knowledge, this is the first published report of a whole-genome sequence of a Quarter Horse and the only horse genome sequenced by next-generation sequencing. At 25X sequence coverage, we estimated that approximately 85-88% of the horse's genome could be genotyped [20]. Our analysis yielded 3.1 million SNPs, 193 thousand INDELs, and 282 CNVs. Despite the fact that approximately 10% of the novel homozygous SNPs likely reflect errors in the reference genome (given that the accuracy of the reference genome is 99.99%) and that our false discovery rate (FDR) for SNP detection is approximately 1.5% (Doan et al. unpublished studies), the genetic variants identified here represent a significant addition to what is currently available for studies in horses. It should be noted that the breeding structure of Quarter Horses and Thoroughbreds, as well as the differences in sequencing technology (i.e., next-generation sequencing of DNA fragments vs. Sanger sequencing of bacterial artificial clones) most likely contributed to the increased amount of genetic variation identified in this study. We suspect that many genetic variants were missed due to the parameters applied during our variant detection (e.g., requiring a minimum of 10X sequence coverage). To minimize this false negative rate (FNR) we provide a list of SNPs and INDELs using a less stringent calling criterion (5X minimum sequence coverage; Additional file 5, **Table S3**). However, caution should be used with these variants as the FDR is expected to increase with a reduced minimum coverage, although not directly proportional to the decrease of the FNR.

*De novo *assembly of reads not mapping to the assembled or un-assembled chromosomes led to the generation of 19.1 Mb of new horse genomic sequence. Our analysis of CNVs in horses and cattle using array comparative genomic hybridization (Doan et al., unpublished studies), as well as studies in the human and mouse show that large (Mb) deletions are common variants in the genome [21-24]. This presence/absence variation [25] is common, and thus we suspect that some percentage of the *de novo *assembled sequence represents sequence missing from the reference Thoroughbred genome due to homozygous deletions.

Functional annotation clustering analysis of genetic variants revealed that pathways for sensory perception, signal transduction, protein processing, cellular process, and immunity and defense were differentially affected by each type of genetic variant (i.e., SNPs, INDELs, and CNVs), suggesting varying degrees of tolerance and selection for genetic variants underlying these biological processes. Genes involved in sensory perception pathways contained most of the genetic variation, primarily SNPs (27%) and CNVs (60%). The observed enrichment in sensory perception genes may be related to selection of the Quarter Horse for a relatively calm disposition [26,27], although this is highly speculative at this point, and will only be determined by future population based-studies. Conversely, these variants could reflect misassemblies in the reference genome or misalignments in the Quarter Horse reads to the reference sequence, as these genes exist as large families with numerous pseudogenes.

## Conclusions

This is the first sequencing of a horse genome by next-generation sequencing and the first genomic sequence of an individual Quarter Horse. The genetic variants identified in this study will be a useful resource for future studies to understand the genetic basis of phenotypic variation and disease in equids.

## Methods

### DNA sample

DNA was isolated from whole blood of a Quarter Horse mare using a standard phenol-chloroform extraction including 2 phenol-chloroform-isoamyl (PCI) steps, followed by rinses with chloroform, isopropanol, and 70% ethanol. The sample was suspended in Qiagen EB buffer (Qiagen Sciences, Germantown, MD). The Texas A&M University Institutional Animal Care and Use Committee approved this study.

### Whole-genome sequencing

For the construction of sequencing libraries, we first sonicated high-quality genomic DNA by pulsing 3X for 15 sec/pulse at 14% using a Sonic Dismembrator 500 (Fisher Scientific, Pittsburg, PA) and purified with an Invitrogen PureLink PCR Kit (Invitrogen, Carlsbad, CA). The DNA was blunt end-repaired, adenylated, and ligated with paired-end adaptors, according to the manufacturer's protocol (Illumina, San Diego CA). The prepared library was resolved on a 2% low range agarose gel and a 2-mm section of DNA was isolated at 271 bp (Qiagen Sciences, Germantown, MD). The library was then enriched according to the manufacturer's protocol (Illumina, San Diego CA). The size and concentration of the sequencing library was determined by PCR, visualization of polyacrylamide gel electrophoresis (PAGE) gels, and through the use of the Agilent 2100 Bioanalyzer DNA kit (Agilent Technologies, San Diego CA). Cluster generation and paired-end sequencing was performed according to the manufacturer's protocols (Illumina) at the AgriLife Genomics and Bioinformatics Center (College Station, TX)

### Sequence alignment

We used the trim function in the CLC Genomics Workbench (CLC Bio, Aarhus, Denmark) using the following parameters: ambiguous limit, 2; ambiguous trim, yes; quality limit, 0.1; quality trim, yes; and, remove 3' nucleotide, no; remove 5' nucleotide, no. The CLC Genomics Workbench Reference Mapping function was used to assemble the trimmed reads to the equCab2 reference assembly using the following parameters: similarity score = 0.8; and, length fraction = 0.5. Paired-end reads were mapped using an insert range of 180-bp to 500-bp and reads mapping to multiple places in the reference were mapped using the random setting. In order to determine the percent of the genome that was mapped by uniquely mapped read, we used the previously stated settings with nonspecific reads being ignored.

### SNP detection

We used the SNP detection function in CLC Genomics Workbench, based on the neighborhood quality standard (NQS) algorithm, using the following parameters: minimum coverage = 5; minimum central base quality = 30; average base quality over a window length of 11 nucleotides = 15; and, minimum allele frequency = 35%. SNPs were filtered by removing those within 10 bp of another variant (both INDELs and SNPs). Also, SNPs located within the pseudo-autosomal region (PAR) were removed [28]. The remaining SNPs were filtered by read-depth to create SNP analyses with minimum depths of 5, 6, 7, 8, 9, and 10.

### INDEL detection

We used the deletion and insertion polymorphism (DIP) function in CLC Genomics Workbench using the following parameters: minimum coverage = 5; minimum allele frequency = 35%; and, maximum expected variations = 2. INDELs were filtered in a similar manner as SNPs, with those near other variants and within the PAR being removed. Analyses were conducted at both 5X and 10X read-depths.

### CNV detection

We used the Control-FREE copy number algorithm (FREEC) program to identify CNVs in the mapped sequence data [16]. We optimized the program for our data using a break-point threshold of -0.0013 and a coefficient of variation of 0.045. Additionally, we removed any CNVs located within 1 Mb of the beginning and end of all chromosomes in order to reduce the potential for erroneous calling.

### Genetic variant annotation and analysis

We re-annotated the variant calls from CLC Genomics using both Galaxy http://galaxy.psu.edu/ and ANNOVAR software programs [29,30]. The join and merge functions in Galaxy were used to annotate the SNPs and INDELs. We used these functions to compare the SNPs to all known SNPs in dbSNP as well as those identified in the reference horse. The ANNOVAR program used the Ensembl annotation database to create an mRNA library, allowing for the determination of amino acid changes. The program was used to determine the locations of all variants within the genome. We also annotated the known SNPs from the reference Thoroughbred genome to determine amino acid changes as well as gene locations for a comparison to the Quarter Horse. The SNPs were divided into groups based on radical and conservative amino acid changes, where radical SNPs result in a difference in polarity or charge when the amino acid was changed while conservative SNPs cause no change in polarity or charge.

The gene lists for each group were converted to human Ensembl gene IDs using Ensembl Biomart. Biological function analysis was performed through the DAVID Functional Annotation Tool, using the default settings [31]. The resulting biological process terms were further grouped by similarities in function to determine enrichment for specific biological processes (Additional file 37, **Table S29**). Statistical significance (p-value) for each enriched group was determined using Fisher's combined probability test with the p-value created from the DAVID Functional Annotation Tool. The enriched genes for each biological process group were compared between the Quarter Horse and reference Thoroughbred using the Fisher's exact test for a 2 × 2 contingency table.

### Analysis of *de novo *assembly of unmapped reads

We created a *de novo *assembly of all reads that did not align to the reference genome (including ChrMt and ChrUn) using de Bruijn Graphs (CLC Genomics). We were able to assemble 8,186,040 of the 12,657,236 unmapped reads into contigs with minimum and average contig lengths of 200 bp and 537 bp, respectively. We performed *de novo *assembly of reads not mapping to either assembled or unassembled chromosomes using the following parameters: similarity = 0.8; length fraction = 0.5; insertion cost = 3; deletion cost = 3; mismatch cost = 3; minimum paired distance = 180 bp; and, maximum paired distance = 500 bp. The resulting 35,540 contigs were further analyzed by BLAST, mapping all complete genomes and chromosomes from RefSeq.

### Sequence data and genetic variants

The Illumina FASTQ data generated from this study has been submitted to the NCBI Sequence Read Archive http://www.ncbi.nlm.nih.gov/sra under accession number SRX110702. The mapped sequences and genetic variants have been submitted to Intrepid Bioinformatics and are available for public viewing http://server1.intrepidbio.com/FeatureBrowser/ngsdatasetrecord/record?ngsrecord=6197673305. A complete list of SNPs, INDELs, and CNVs are listed in Additional files 5, 6, and 24, Tables S3, S4 and S24.

## Competing interests

The authors declare that they have no competing interests.

## Authors' contributions

RD generated the sequencing libraries and analyzed the data. NG and CJ sequenced the libraries and analyzed the data. RD, NDC, JS and SVD conceived the study and wrote the manuscript. All authors read and approved the final manuscript.

## Supplementary Material

Additional file 1 **Distribution of paired-end sequencing library insert length**. Figure indicating distribution of library insert length and number of sequence reads generated per length. Click here for file

Additional file 2 **Sequencing reaction statistics**. Table of raw summary statistics of each lane of Illumina sequencing. Click here for file

Additional file 3 **Detailed overview of mapping results**. Table of total sequence reads (paired and single end), the number of mapped reads and bp to assembled and unassembled chromosomes. Click here for file

Additional file 4 **Average depth of coverage of assembled chromosomes**. Figure of average mapping read coverage of each chromosome. Click here for file

Additional file 5 **5X filtered SNPs and INDELS identified by whole genome sequencing**. Table listing chromosomal coordinates of SNPs and INDELs identified using a minimum sequence read depth coverage of 5X. Click here for file

Additional file 6 **10X filtered SNPs and INDELS identified by whole genome sequencing**. Table listing chromosomal coordinates of SNPs and INDELs identified using a minimum sequence read depth coverage of 10X. Click here for file

Additional file 7 **Loss of SNPs by post-filtering**. Table describing the number of SNPs and INDELs removed by post-filtering. Click here for file

Additional file 8 **Comparison of minimum depth of coverage SNP filters**. Total number of (A) SNPs and (B) non-synonymous SNP remaining after increasing the minimum sequence read depth of coverage. Proportion of homozygous and heterozygous (C) SNPs and (D) non-synonymous SNPs lost by increasing minimum sequence read depth of coverage. Click here for file

Additional file 9 **Annotation of SNPs with minimum read depth coverage of 5X**. Table describing the genic annotation of SNPs identified at 5X coverage. Click here for file

Additional file 10 **Annotation of SNPs with minimum read depth coverage of 5X relative to the Ensembl gene annotation**. Table describing the intergenic and genic location of SNPs identified at 5X coverage. Click here for file

Additional file 11 **Genes affected by SNPs with minimum read depth coverage of 5X**. Table describing the number of genes identified with SNPs, including the annotation of the SNPs within the genetic elements. Click here for file

Additional file 12 **Overview of SNP annotation with minimum read depth coverage of 5X**. Table summarizing the number and annotation of SNPs with minimum read depth coverage of 5X. Click here for file

Additional file 13 **Annotation of SNPs with minimum read depth coverage of 6X**. Table describing the genic annotation of SNPs identified with minimum read depth coverage of 6X coverage. Click here for file

Additional file 14 **Annotation of SNPs with minimum read depth coverage of 6X relative to the Ensembl gene annotation**. Table describing the intergenic and genic location of SNPs identified with minimum read depth coverage of 6X coverage. Click here for file

Additional file 15 **Annotation of SNPs with minimum read depth coverage of 7X**. Table describing the genic annotation of SNPs identified with minimum read depth coverage of 7X coverage. Click here for file

Additional file 16 **Annotation of SNPs with minimum read depth coverage of 7X relative to the Ensembl gene annotation**. Table describing the intergenic and genic location of SNPs identified with minimum read depth coverage of 7X coverage. Click here for file

Additional file 17 **Annotation of SNPs with minimum read depth coverage of 8X**. Table describing the genic annotation of SNPs identified with minimum read depth coverage of 8X coverage. Click here for file

Additional file 18 **Annotation of SNPs with minimum read depth coverage of 8X relative to the Ensembl gene annotation**. Table describing the intergenic and genic location of SNPs identified with minimum read depth coverage of 8X coverage. Click here for file

Additional file 19 **Annotation of SNPs with minimum read depth coverage of 9X**. Table describing the genic annotation of SNPs identified with minimum read depth coverage of 9X coverage. Click here for file

Additional file 20 **Annotation of SNPs with minimum read depth coverage of 9X relative to the Ensembl gene annotation**. Table describing the intergenic and genic location of SNPs identified with minimum read depth coverage of 9X coverage. Click here for file

Additional file 21 **Annotation of SNPs with minimum read depth coverage of 10X**. Table describing the genic annotation of SNPs identified with minimum read depth coverage of 10X coverage. Click here for file

Additional file 22 **Annotation of SNPs with minimum read depth coverage of 10X relative to the Ensembl gene annotation**. Table describing the intergenic and genic location of SNPs identified with minimum read depth coverage of 10X coverage. Click here for file

Additional file 23 **Genes affected by SNPs with minimum read depth coverage of 10X**. Table describing the number of genes identified with SNPs, including the annotation of the SNPs within the genetic elements. Click here for file

Additional file 24 **Imprinted genes containing SNPs in coding regions**. List of known imprinted genes in humans containing coding SNPs in Quarter Horse. Click here for file

Additional file 25 **Annotation of INDELs with minimum read depth coverage of 5X**. Click here for file

Additional file 26 **Annotation of INDELs with minimum read depth coverage of 10X**. Table describing genic annotation and location of INDELs with minimum read depth coverage of 10X. Click here for file

Additional file 27 **Distribution of total number of INDELs by length**. Plot showing the number of INDELS by length identified with minimum read depth coverage of 10X. Click here for file

Additional file 28 **Coordinates of CNVs**. Table describing the location of CNVs identified by analysis of read depth. Click here for file

Additional file 29 **Identification of a loss within an olfactory gene cluster by sequencing read-depth**. Plot of read depth indicating a copy number loss within olfactory gene cluster. Click here for file

Additional file 30 **Identification of a homozygous deletion by read-depth coverage**. Plot of read depth indicating a homozygous deletion. Click here for file

Additional file 31 **Biological process enrichments and associated p-values for SNPs, INDELS, and CNVs with minimum read depth coverage of 10X**. Statistical analyses of biological process enrichments for SNP, INDELS, and CNVs with minimum read depth coverage of 10X. Click here for file

Additional file 32 **Biological process enrichment analysis of conserved and radical non-synonymous SNPs**. Statistical analyses of biological process enrichments for conserved and radical SNPs with minimum read depth coverage of 10X. Click here for file

Additional file 33 **Annotation of SNPs in the reference Thoroughbred genome**. Table describing genic annotation and location of known SNPs in the reference Thoroughbred genome. Click here for file

Additional file 34 **Comparison of biological processes affected by SNPs in the Quarter Horse and Thoroughbred genomes**. (A) BP enrichment of non-synonymous homozygous SNPs and (B) heterozygous SNPs in the Quarter Horse genome. (C) BP enrichment of non-synonymous heterozygous SNPs in the Thoroughbred genome. (D) Number of genes containing non-synonymous SNPs within each BP pathway. Click here for file

Additional file 35 **Biological process enrichments and associated p-values for SNP comparison between Quarter Horse and reference Thoroughbred with minimum coverage of 10 reads**. Comparison and statistical analysis of biological process enrichments between Quarter Horse and reference Thoroughbred. Click here for file

Additional file 36 **Statistical analysis of enriched genes in the Quarter Horse and reference Thoroughbred**. Statistical analysis of comparison between enriched genes in the Quarter Horse and reference Thoroughbred. Click here for file

Additional file 37 **Biological process terms and classification**. All terms used for biological processes enrichment and their associated category. Click here for file
