# Supplementary figures and images for: Whole-Genome sequencing and genetic variant analysis of a quarter Horse mare
Source: BMC Genomics. 2012 Feb 17;13:78. doi: 10.1186/1471-2164-13-78 (PMC3309927; doi:10.1186/1471-2164-13-78)

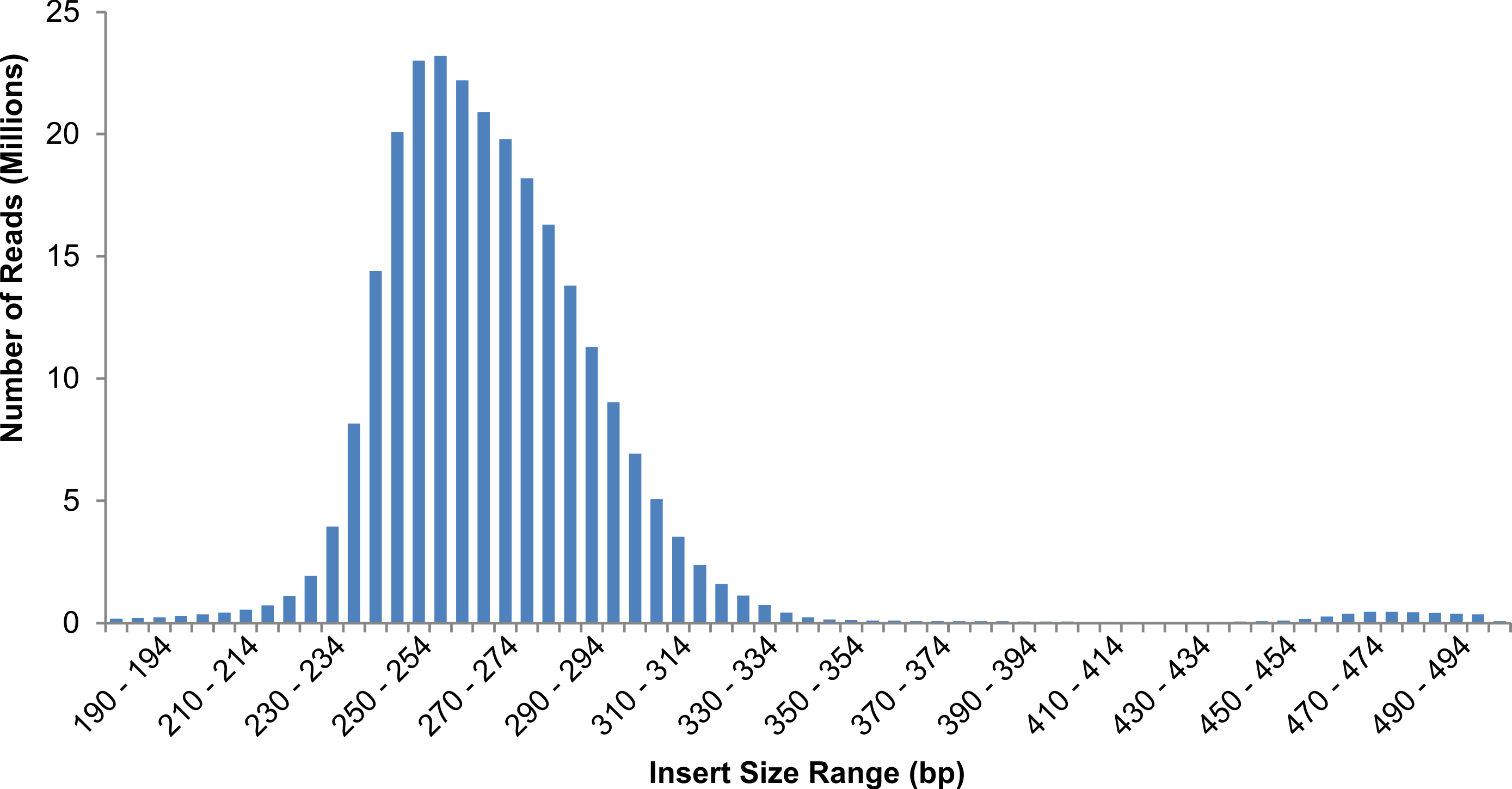

Supplement: Additional file 1 — Distribution of paired-end sequencing library insert length. Figure indicating distribution of library insert length and number of sequence reads generated per length. [file 1471-2164-13-78-S1.TIFF]

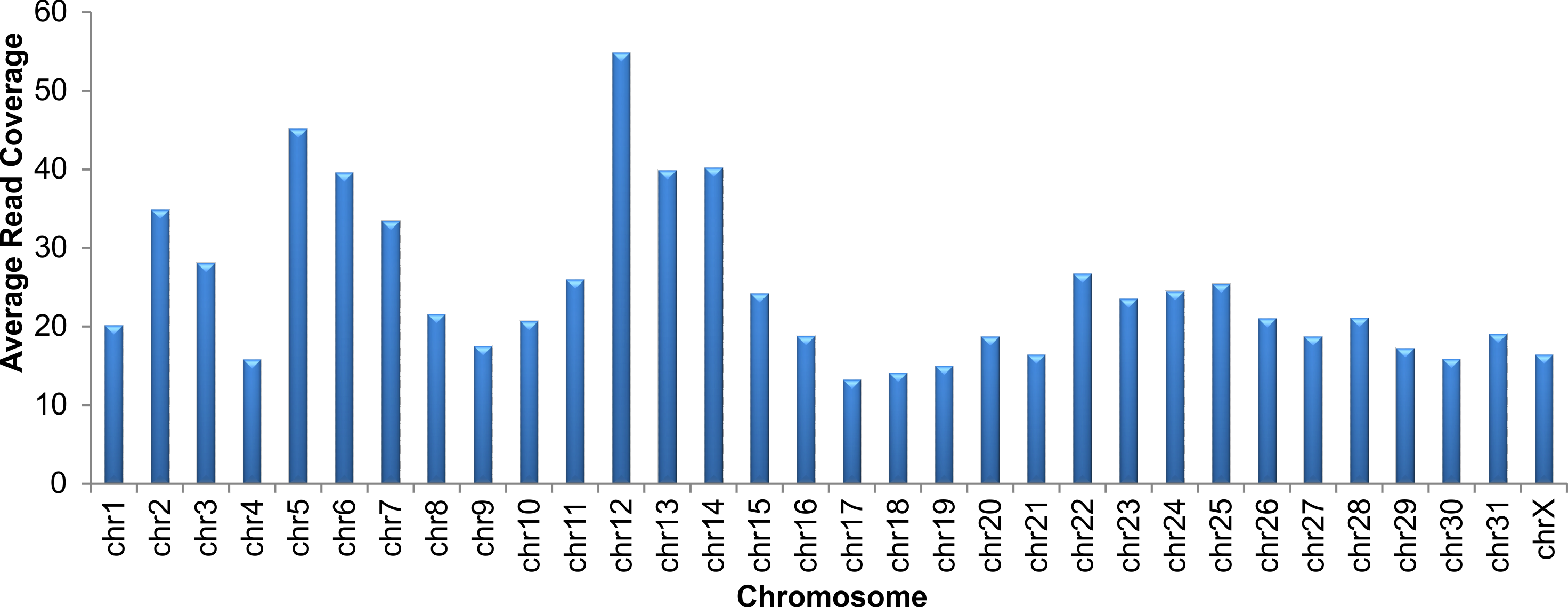

Supplement: Additional file 4 — Average depth of coverage of assembled chromosomes. Figure of average mapping read coverage of each chromosome. [file 1471-2164-13-78-S4.TIFF]

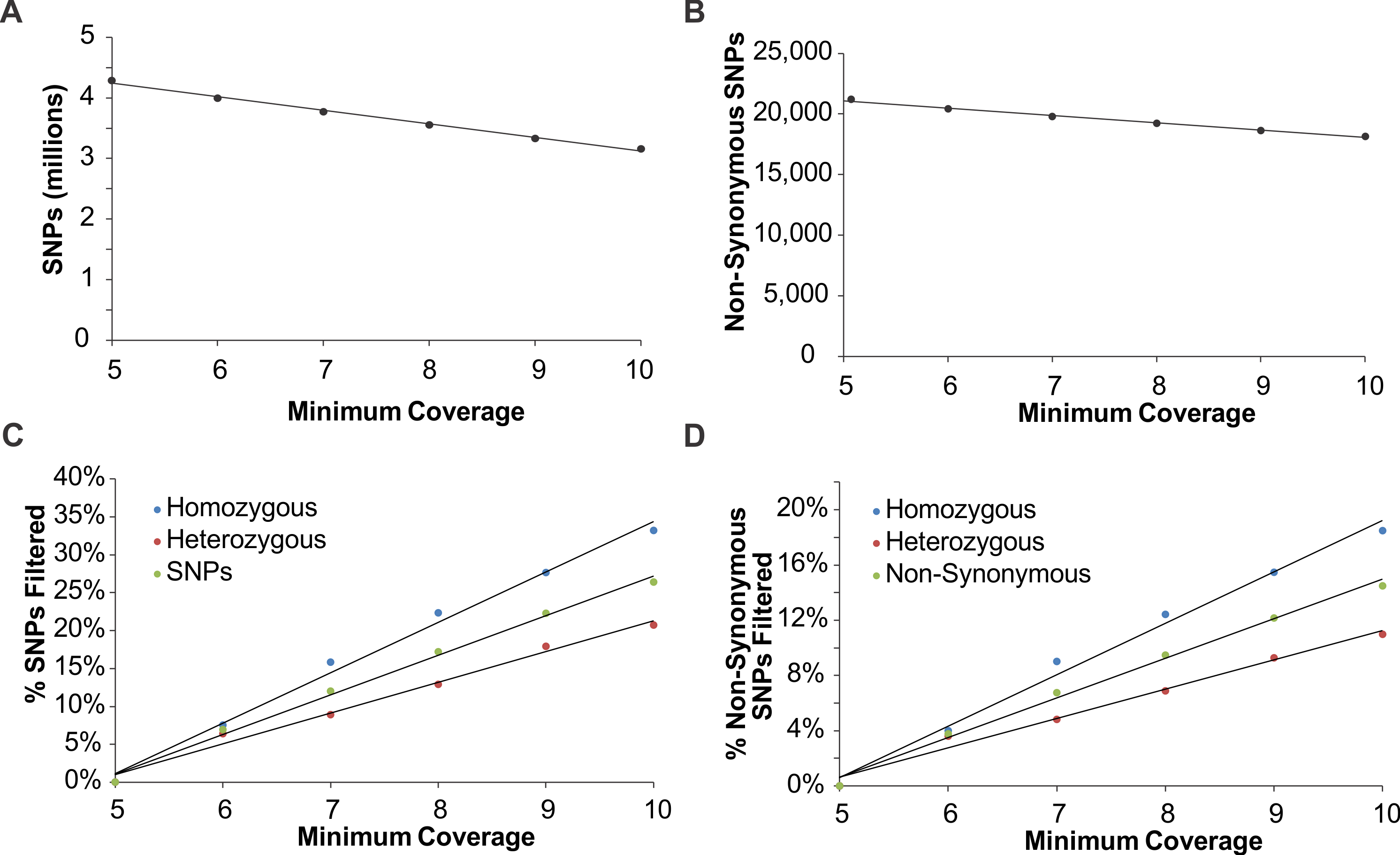

Supplement: Additional file 8 — Comparison of minimum depth of coverage SNP filters. Total number of (A) SNPs and (B) non-synonymous SNP remaining after increasing the minimum sequence read depth of coverage. Proportion of homozygous and heterozygous (C) SNPs and (D) non-synonymous SNPs lost by increasing minimum sequence read depth of coverage. [file 1471-2164-13-78-S8.TIFF]

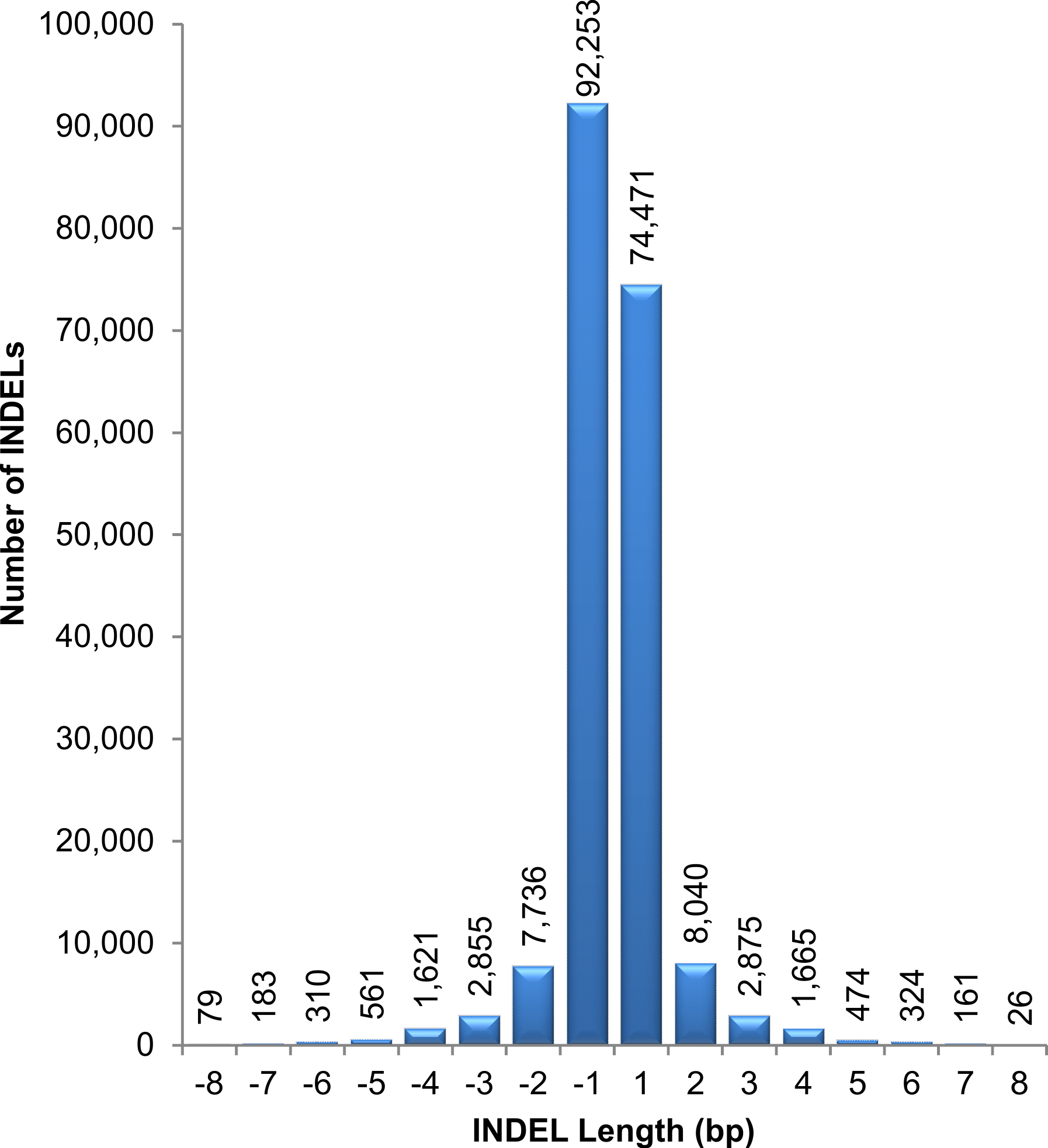

Supplement: Additional file 27 — Distribution of total number of INDELs by length. Plot showing the number of INDELS by length identified with minimum read depth coverage of 10X. [file 1471-2164-13-78-S27.TIFF]

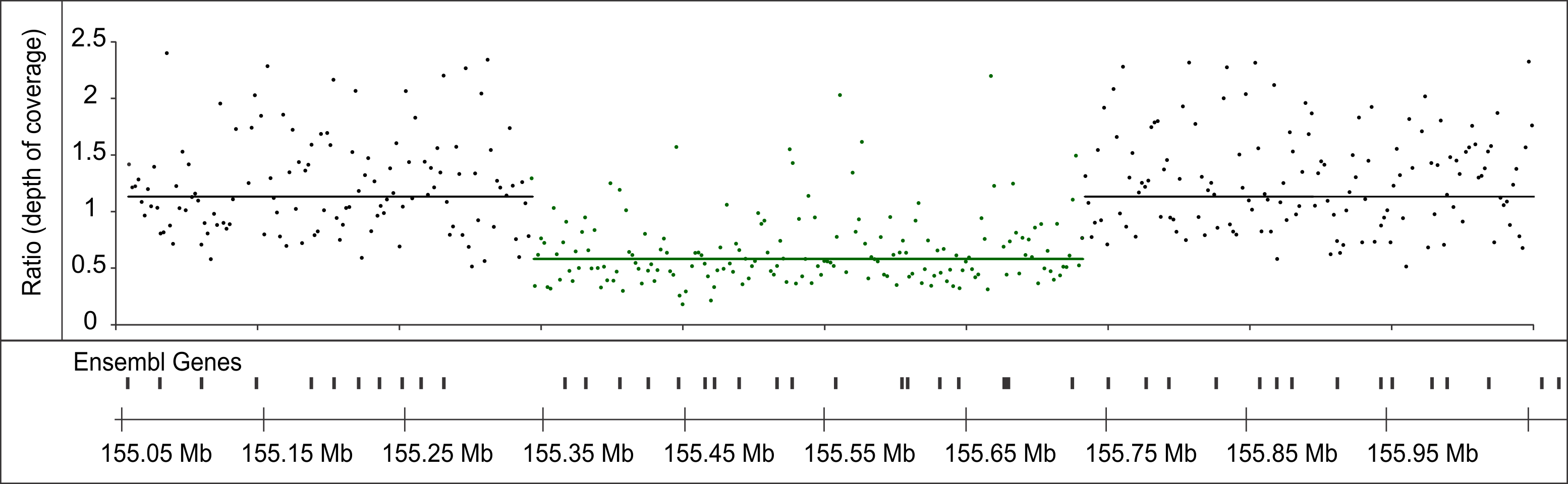

Supplement: Additional file 29 — Identification of a loss within an olfactory gene cluster by sequencing read-depth. Plot of read depth indicating a copy number loss within olfactory gene cluster. [file 1471-2164-13-78-S29.TIFF]

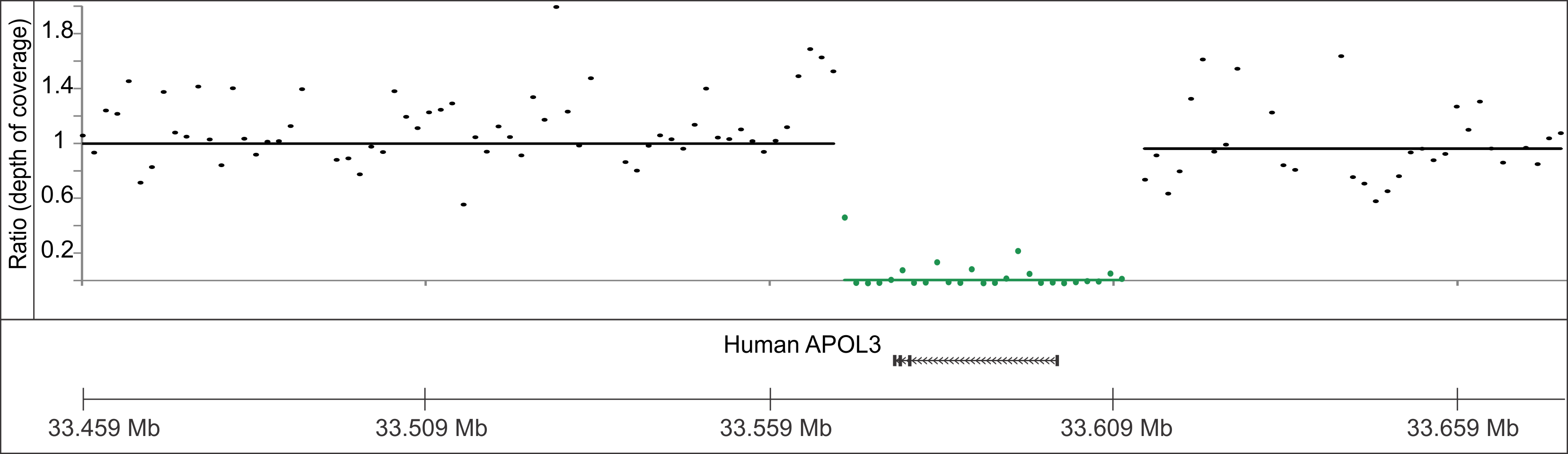

Supplement: Additional file 30 — Identification of a homozygous deletion by read-depth coverage. Plot of read depth indicating a homozygous deletion. [file 1471-2164-13-78-S30.TIFF]

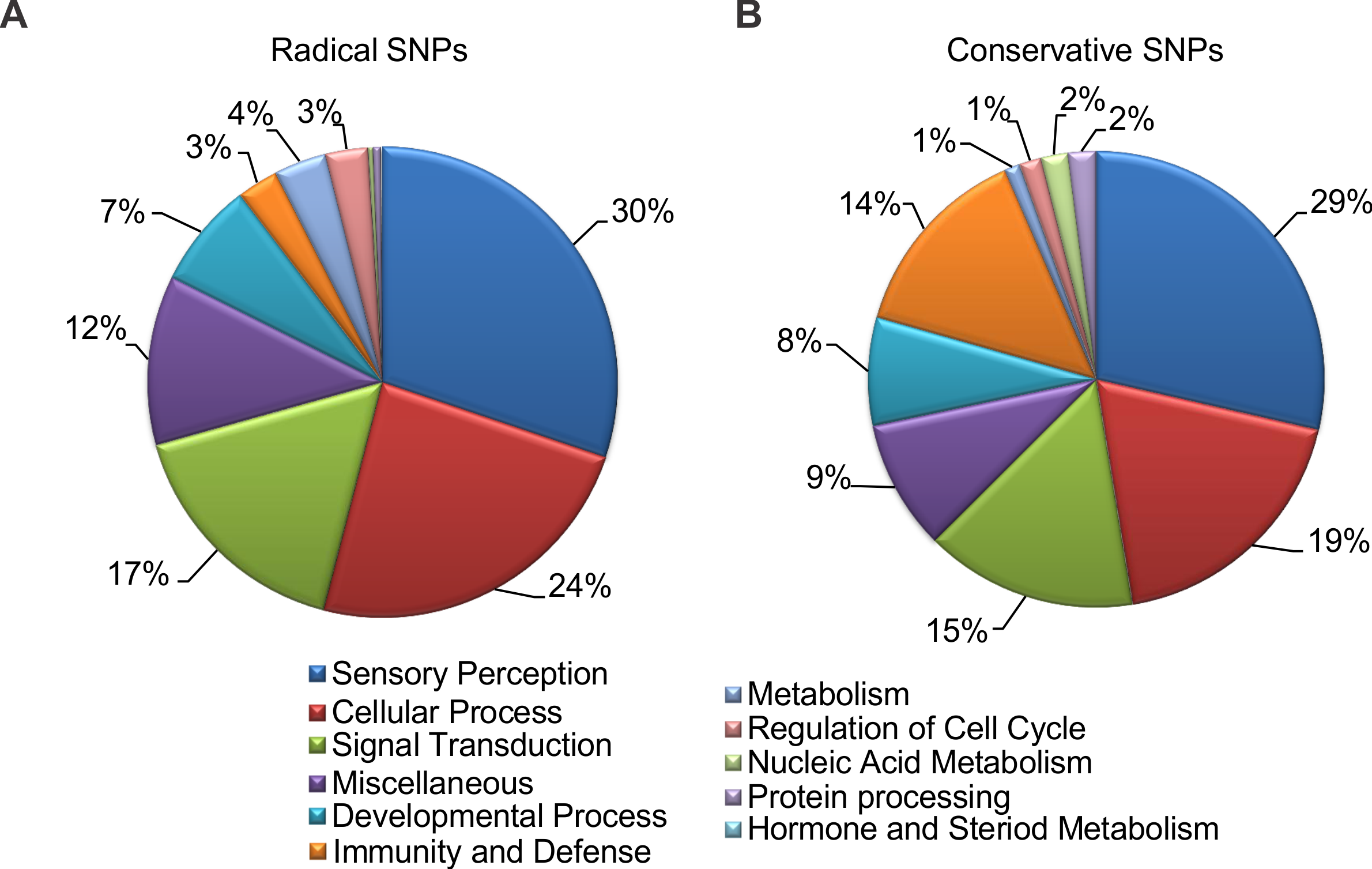

Supplement: Additional file 32 — Biological process enrichment analysis of conserved and radical non-synonymous SNPs. Statistical analyses of biological process enrichments for conserved and radical SNPs with minimum read depth coverage of 10X. [file 1471-2164-13-78-S32.TIFF]

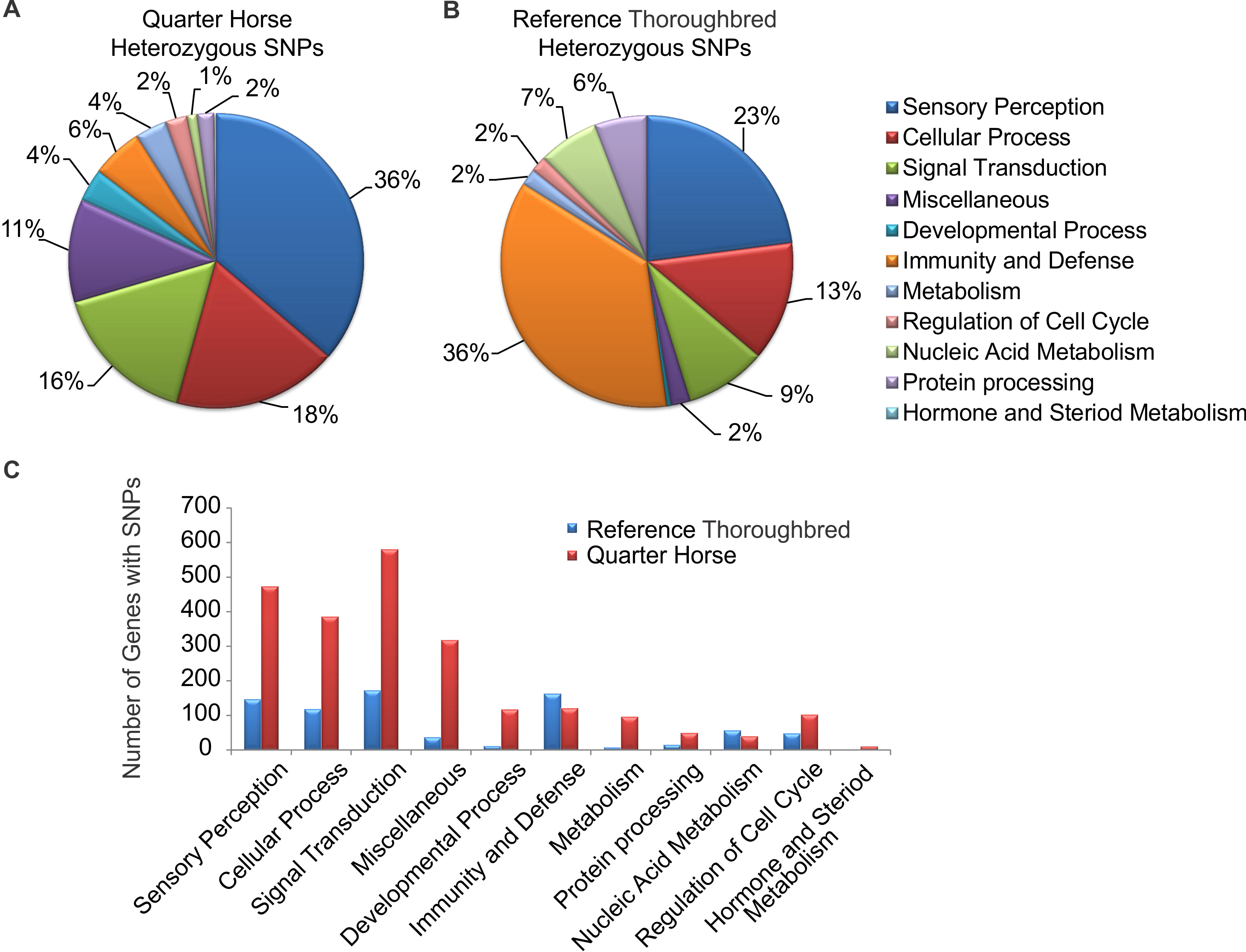

Supplement: Additional file 34 — Comparison of biological processes affected by SNPs in the Quarter Horse and Thoroughbred genomes. (A) BP enrichment of non-synonymous homozygous SNPs and (B) heterozygous SNPs in the Quarter Horse genome. (C) BP enrichment of non-synonymous heterozygous SNPs in the Thoroughbred genome. (D) Number of genes containing non-synonymous SNPs within each BP pathway. [file 1471-2164-13-78-S34.TIFF]
